# Structural Features Determining the Intestinal Epithelial Permeability and Efflux of Novel HIV-1 Protease Inhibitors

**DOI:** 10.1002/jps.22570

**Published:** 2011-04-13

**Authors:** Lucia Lazorova, Ina Hubatsch, Jenny K Ekegren, Johan Gising, Daisuke Nakai, Noha M Zaki, Christel A S Bergström, Ulf Norinder, Mats Larhed, Per Artursson

**Affiliations:** 1 Department of Pharmacy, Uppsala UniversitySE-751 23 Uppsala, Sweden; 2 The Uppsala University Drug Optimization and Pharmaceutical Profiling Platform (UDOPP), Uppsala UniversitySE-751 23 Uppsala, Sweden; 3 Department of Medicinal Chemistry, Organic Pharmaceutical Chemistry, BMC, Uppsala UniversitySE-751 23 Uppsala, Sweden; 4 AstraZeneca, Research and DevelopmentInnovative Medicines B218, SE-15185 Södertälje, Sweden

**Keywords:** Absorption, ADME, Caco-2 cells, Drug design, Drug transport, HIV/AIDS, Intestinal absorption, Passive diffusion/transport, P-glycoprotein, Permeability

## Abstract

The primary aim of this study was to identify structural features that alter the intestinal epithelial permeability and efflux in a series of novel HIV-1 protease inhibitors (PIs). Eleven PIs were selected containing a tertiary alcohol in a transition-state mimicking scaffold, in which two substituents (R_1_ and R_2_) were varied systematically. Indinavir was selected as a reference compound. The apical-to-basolateral permeability was investigated in 2/4/A1 and Caco-2 monolayers. In addition, the basolateral-to-apical permeability was investigated in the Caco-2 monolayers and the efflux ratios were calculated. The absence of active drug transport processes in 2/4/A1 cells allowed identification and modeling of structural elements affecting the passive permeability. For instance, small aromatic R_1_ substituents and a small (bromo-) R_2_ substituent were associated with a high passive permeability. Efflux studies in Caco-2 cells indicated that amide-substituted neutral hydrophobic amino acids, such as valine and leucine, in the R_1_ position, reduced the apical-to-basolateral transport and enhanced the efflux. We conclude that our investigation revealed structural features that alter the intestinal epithelial permeability and efflux in the series of PIs and hope that these results can contribute to the synthesis of PIs with improved permeability and limited efflux properties. © 2010 Wiley-Liss, Inc. and the American Pharmacists Association J Pharm Sci 100:3763–3772, 2011

## INTRODUCTION

Drug absorption across the human intestinal wall occurs predominantly via passive transcellular diffusion. Also for many drugs that are substrates for uptake and efflux of transport proteins in the intestinal epithelium, passive transport often contributes significantly to the total transport, in particular at clinical doses.^1^ This simplifies the predictions of intestinal drug permeability and absorption, as a result of which fairly good predictions have been obtained without the consideration of any active transport mechanisms.^2^,^3^ One predictive model is 2/4/A1, a conditionally immortalized rat intestinal epithelial cell line that forms tight cell monolayers with a permeability that is comparable to that of the human jejunum.^4^,^5^ When grown on permeable supports, the permeability to registered drugs is quantitatively comparable to that of the human intestine^6^,^7^ despite the fact that 2/4/A1 cells do not have functional drug transporting proteins such a P-glycoprotein (P-gp).^8^ However, whether this cell line would also prove to be useful for permeability profiling in a drug discovery setting had not, as yet, been investigated.

For some drug classes, including HIV-1 protease inhibitors (PIs), active transport mechanisms, such as P-gp-mediated efflux, are common. The coexistence of active and passive transport mechanisms may complicate both *in vitro* and *in silico* predictions of intestinal permeability in models such as 2/4/A1. Caco-2 cell monolayers represent the most commonly used cell culture model for *in vitro* studies of intestinal permeability, and they afford the opportunity to investigate both passive and active transport processes.^9^–^11^ Caco-2 monolayers express many active transport mechanisms of the human small intestine, including functional efflux proteins, such as P-gp, and therefore they are commonly used to identify compounds with a high drug efflux.^12^,^13^

Drug-like compounds in drug discovery settings have not generally been optimized with regard to properties such as the intestinal permeability and transport, although rough predictions can be made from molecular descriptors.^14^ In this contribution, therefore, we investigated the *in vitro* permeability and transport of a series of HIV-1 PIs that are structural variants of registered PIs, including indinavir. This series was chosen because PIs are associated with poor bioavailability, and therefore there is a need to identify molecular determinants of features such as active and passive drug transport across the intestinal epithelium.^15^–^17^

More specifically, we studied a new class of compounds comprising a shielded, tertiary alcohol as part of the transition-state mimicking scaffold.^18^–^21^ In these inhibitors, the polar hydroxyl group may form intramolecular hydrogen bonds and is well masked by the surrounding carbon skeleton, features often used to improve the membrane permeation ability of organic compounds.^22^–^24^ In the reported *P*1′ *para*-functionalized series, wherein the main difference is in the length of the carbon core structure and in the *P*2 side chain, highly potent inhibitors were obtained with *K*_i_ values below 10 nM and cellular half maximal effective concentration (EC_50_) activities below 0.50 µM. To our delight, good metabolic stability in microsome preparations and good Caco-2 cell membrane permeability were recorded for several compounds. However, despite that, low *K*_i_ values were obtained with *P*1′ *meta*-substituted inhibitors in the cell-free assays; we could not explain why these compounds were devoid of activity in the cell assay.

Thus, with the intention of investigating this issue, we selected a subset of PI analogues that was representative of the differing inhibitory potential in cell-free assays, in comparison with cell assays, and investigated their permeability in 2/4/A1 and Caco-2 cell monolayers. As the lower activity in the cell assays could be due to efflux by ABC transporters such as P-gp, we also investigated their transport in the secretory direction across Caco-2 cells. Through this approach, we could both evaluate the performance of 2/4/A1 cells as an alternative cell model for permeability screening and investigate whether the passive membrane permeability and/or drug efflux could explain the observed reduction of PIs activity in the cell-based assays. Structural features that alter passive permeability and efflux by ABC transporters could also be identified.

## MATERIALS AND METHODS

Unless otherwise stated, all culture media and supplements were purchased from Invitrogen AB (Lidingö, Sweden). Hanks' balanced salts solution (HBSS), epidermal growth factor (EGF), insulin-like growth factor-1, insulin, human transferrin and selenous acid premix, water-soluble dexamethasone, extracellular matrix (ECM) protein gel, 4-(2-hydroxyethyl)-1-piperazineethanesulfonic acid (HEPES), bovine serum albumin (BSA), Triton-X100, dimethyl sulfoxide (DMSO), and acetonitrile were purchased from Sigma–Aldrich (Stockholm, Sweden). [^14^C] mannitol was purchased from PerkinElmer Sverige AB, (Upplands Väsby, Sweden) and the phosphate-buffered saline was purchased from (Medicago, Sweden).

### 2/4/A1 Cell Culture

2/4/A1 cells were conditionally immortalized with a pZipSVtsa58 plasmid containing a temperature-sensitive mutant of the SV40 large T antigen. The cells were expanded at 33°C in RPMI (Roswell Park Memorial Institute Medium) 1640 medium supplemented with 5% fetal calf serum, 2 mM l-glutamine, and 20 ng/mL EGF. At passage numbers 32 to 45, 2/4/A1 cells were seeded on permeable polycarbonate filter supports (0.45-µm pore size, 12-mm diameter; Transwell Costar, Sigma–Aldrich), freshly coated with an ECM protein gel (16 µg/cm^2^) at a density of 100,000 cells/cm^2^. NCTC-109, with the following additives, was used for the filter-grown cells: 2 mM l-glutamine, 10 µM dexamethasone (water soluble), 10 mM HEPES, 0.1% BSA, penicillin (100 U/mL), and streptomycin (100 µg/mL). The cells were allowed to grow at 39°C for 6–7 days before the monolayers were used in drug transport experiments. At this time, the cell monolayers used in this study had a mannitol permeability of less than 6 × 10^−6^ cm/s and a transepithelial electrical resistance (TEER) of more than 35 ohm × cm^2^.

### Caco-2 Cell Culture

Caco-2 cells originating from a human colorectal carcinoma were obtained from American Type Culture Collection (Manassas, Virginia) and were cultivated as described previously in detail.^13^ In brief, Caco-2 cells were seeded at a density of 330,000 cells/cm^2^ on permeable polycarbonate filter supports (0.45-µm pore size, 12-mm diameter; Transwell Costar, Sigma–Aldrich) in Dulbecco's modified Eagle's medium supplemented with 10% fetal calf serum, 1% Minimum Essential Medium nonessential amino acids, penicillin (100 U/mL), and streptomycin (100 µg/mL). Caco-2 cells were used at passage numbers 95 through 105. The Caco-2 cell monolayers were chosen for transport experiments between 21 and 28 days after seeding. At this time, the cell monolayers used in this study had a mannitol permeability of less than 0.3 × 10^−6^ cm/s and a TEER of more than 240 ohm × cm^2^.

### Integrity Studies

The integrity of the 2/4/A1 and Caco-2 monolayers before the experiments and after the exposure of each of the compounds under investigation (observed solubility in HBSS, pH 7.4, >5 µM) was determined using the paracellular marker molecule [^14^C] mannitol (specific activity 2.176 GBq/mmol) and by measuring the TEER, as described elsewhere.^13^

Each compound was dissolved in DMSO as a 1 mM stock solution. Before the experiments, they were further diluted in HBSS containing 25 mM HEPES, pH 7.4, to a final concentration of 5 µM.

The transepithelial transport of [^14^C] mannitol across the epithelial cell monolayer was assessed by the apparent permeability coefficient, *P*_app_, according to Eq. (1):


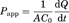
(1)

*Q* is the amount of [^14^C] mannitol transported into the basolateral chamber, *t* is the elapsed time, *A* is the area available to transport (1.131 cm^2^), and *C*_0_ is the initial concentration of [^14^C] mannitol. The *P*_app_ was expressed in centimeter per second.

The PIs investigated in this study did not affect the integrity of the 2/4/A1 cell and Caco-2 monolayers, as measured by transepithelial transport of [^14^C] mannitol. Thus, [^14^C] mannitol permeability was not significantly different to control monolayers after incubation with the PIs.

### Transport Experiments In 2/4/A1 And Caco-2 Monolayers

The investigation of the transport of the compounds **1–11** and indinavir was performed in accordance with published protocols,^8^,^13^,^25^ with the modification of 1% BSA being included in all receiver chambers to improve the mass balance.^26^ Briefly, filters with mature 2/4/A1 and Caco-2 monolayers were washed with prewarmed HBSS, pH 7.4, for 15 min, whereupon the HBSS was replaced with prewarmed donor solutions of the compounds under investigation (5–10 µM). The transport experiments were carried out in the apical-to-basolateral (a–b) direction in the 2/4/A1 monolayers and both the apical-to-basolateral (a–b) and basolateral-to-apical (b–a) directions in Caco-2 monolayers. The monolayers were incubated at 37°C, and intense orbital stirring (500 rpm) was applied to reduce the effect of the unstirred water layer adjacent to the cell monolayers. The donor and receiver compartments were sampled at regular time intervals (generally 5, 15, and 30 min for highly permeable substances; and 5, 45, and 90 min for substances with intermediate and low permeability). The final sampling from the donor chamber was used to calculate the mass balance of the compound. In general, a mass balance of 80% was obtained. Donor samples were diluted 10 times in HBSS–acetonitrile (33:67, v/v), and the receiver samples were diluted in two volumes of acetonitrile. Precipitated BSA was removed by centrifugation at 12,500g at 4°C for 15 min. Thereafter, additional dilution in HBSS–acetonitrile (33:67, v/v) was performed when necessary. The samples were analyzed by liquid chromatography–tandem mass spectrometry (LC–MS/MS) immediately after the transport experiments.

Permeability (*P*_app_) values (cm/s) were calculated in accordance with Eq. (2) (which applies to nonsink conditions), where *C*_R_(*t*) is the time-dependent drug concentration in the receiver compartment, *M* is the amount of drug in the system, *V*_D_ and *V*_R_ are the volumes of the donor and receiver compartments, respectively, *A* is the surface area of the filter (cm^2^), and *t* is the time from the start of the interval.^27^


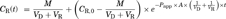
(2)

### LC–MS/MS Analysis

The analysis of compounds **1–11** and indinavir was performed on a Thermo Finnigan TSQ Quantum Discovery triple-quadrupole mass spectrometer (Thermo Scientific, Waltham, Massachusetts) equipped with a Finnigan Surveyor autosampler and high performance liquid chromatography (HPLC) pump. Chromatographic separation was performed on a ReproSil-Pur C8 (50 × 3 mm^2^, 5 u) analytical column supplied by Dr A. Masch. The HPLC was operated at a flow rate of 200 µL/min with two mobile phases (A and B): A = 0.1% formic acid/5% acetonitrile (v/v) and B = 0.1% formic acid/100% acetonitrile. Typically, the following gradients were applied: %B/time (min); 50/0–2.5, 95/2.5–4, 50/4–7 or 20/0–0.5, 95/0.5–5.5, and 20/5.5–9. The sample injected was 10 µL and all samples contained one volume of sample and two volumes of acetonitrile. The peak areas obtained in the chromatograms were integrated automatically by the mass spectrometry software (Xcalibur 1.4, Thermo Scientific, Waltham, Massachusetts). The concentration was calculated from linear regression of standard samples with Graphpad Prism 4 (Graphpad Software Inc., La Jolla, California).

### Molecular Descriptors

Three-dimensional molecular structures were generated from SMILES representations for the calculations of the topological polar surface area (TPSA), C log *P*, log *D*, log acid dissociation constant (p*K*a), number of rotatable bonds, number of donor atoms for H bonds, number of acceptor atoms for H bonds, and other molecular descriptors, using Corina version 3.0 (Molecular Networks, Erlangen, Germany). These were used as the input for the molecular descriptor calculation, which was performed with DragonX version 1.4 (Talete, Milano, Italy) and ADMETPredictor version 5.0 (Simulations Plus, Lancaster, California). For the computational modeling, the compounds in this investigation were described both in terms of their entire structures and as consisting of a common core with two substituents, R_1_ and R_2_ (Table 1). Dragon descriptors were calculated for the entire molecule, and for the substituent positions R_1_ and R_2_. In total, 141 and 282 descriptors were used.

**Table 1:** Structures, Molecular Properties, Activity, Permeability, and Efflux of the HIV-1 Protease Inhibitors

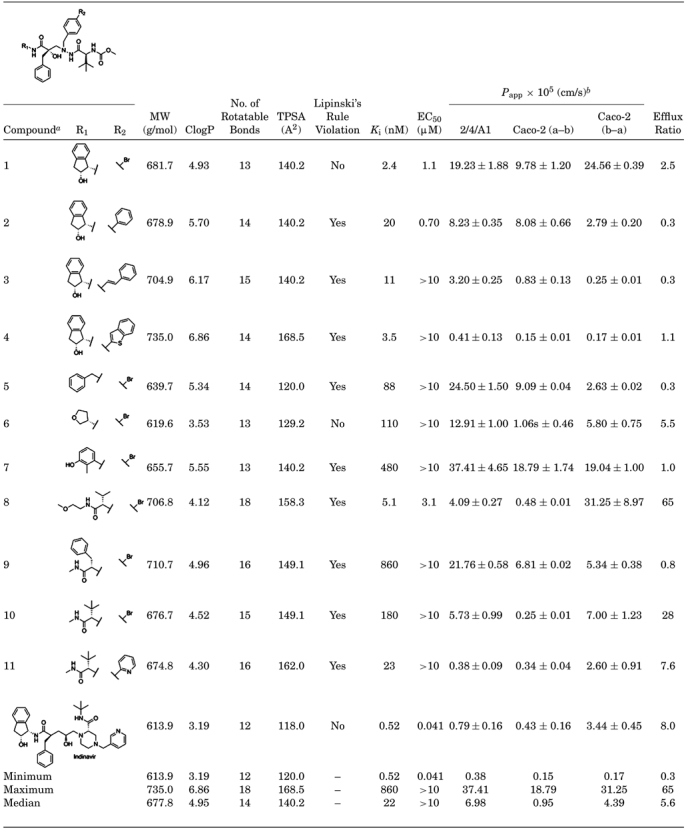

^*a*^The experimental procedures and characterization have been reported previously in the following references: **1–4**^28^, **5–10**^19^ and for the experimental details for **11**, see the supplementary information. The molecular descriptors were calculated as described in *Materials and Methods*.

^*b*^The permeability coefficients were determined in the apical-to-basolateral (a–b) direction in 2/4/A1 cell monolayers and in the apical-to-basolateral (a–b) and/or basolateral-to-apical (b–a) direction in Caco-2 cell monolayers, respectively, as described in *Materials and Methods*. The permeability coefficients are presented as the mean values ± SD, *n* = 3.

TPSA, topological polar surface area.

### Synthesis And Biological Evaluation Of HIV-1 Protease Inhibitors

The synthesis, characterization, and biological evaluation (*K*_i_, EC_50_) of HIV-1 PIs have been reported previously for compounds **1–4**,^28^ and **5–10**,^19^ (Table 1). The synthesis, characterization, and biological evaluation of methyl (S)-1-(2-((S)-2-benzyl-3-((S)-3,3-dimethyl-1-(methylamino)-1-oxobutan-2-ylamino)-2-hydroxy-3-oxopropyl)-2-(4-(pyridin-2-yl)benzyl)hydrazinyl)-3,3-dimethyl-1-oxobutan-2-ylcarbamate (**11**) are described in the supplementary information.

### Statistics

The permeability results are expressed as the mean values ± SD of three monolayers. The experiments were repeated on at least two independent occasions. One-way analysis of variance was used to compare the means, and a 95% probability was considered to be significant.

The partial least-squares projection to latent structures (PLS) analyses were performed in version 12.0 of the Simca-P software (Umetrics AB, Umeå, Sweden). The data were mean centered and scaled to unit variance. A variable selection procedure was applied. Variables exhibiting low variable importance were removed. The aim of the variable selection was to maintain predictivity and to increase the robustness of the models, as well as to decrease the complexity of the models and to facilitate interpretation by removing information that was not directly related to the response variable (i.e., noise). The accuracy of the PLS models was judged by the *R*^2^ and the square root of the variance of the residuals. The models were validated by fourfold cross-validated *R*^2^ (*Q*^2^) (see supplementary information). Transport across 2/4/A1 cells was analyzed and modeled as the square root of the original *P*_app_ values.

## RESULTS

The calculated molecular descriptors indicated that all compounds in the series **(1–11** and indinavir) were uncharged at physiological pH and had rather high molecular weights (>613.9 g/mol), lipophilicities (>3.19), rotatable bonds (>12), and TPSAs (>120.0 Å^2^), as shown in Table 1. Thus, the compound series had molecular properties that are generally considered to be disadvantageous for the membrane permeability and drug absorption. For instance, only three of the compounds, including indinavir, did not violate Lipinski's rule of five, whereas permeability experiments indicated that all compounds had intermediate-to-high permeabilities in the absorptive direction in both cell models (Table 1). Furthermore, most compounds had a TPSA of larger than 120–140 Å^2^ (range 120.0–168.5 Å^2^), which was the value originally proposed the interval in which the cutoff for orally available drugs should lie.^29^,^30^ The rather high membrane permeability in spite of this violation can, possibly, be explained by sterical protection of and/or intramolecular hydrogen bond formation by the tertiary hydroxyl group (which has a polar surface area of 20 Å^2^) because these effects are not accounted by the TPSA or other relevant molecular descriptors used in this study.

The definitions of high, intermediate, and low permeabilities in the present paper are consistent with those presented by Matsson et al.,^7^ illustrating the reproducibility of these cell models in our laboratory. Thus, in 2/4/A1 cells, highly permeable compounds were defined as those having a permeability coefficient of more than 40 × 10^−6^ cm/s, whereas a permeability range of 8–40 × 10^−6^ cm/s defined compounds with intermediate permeability. Low permeable compounds had permeability coefficients of less than 8 × 10^−6^ cm/s. The corresponding values for Caco-2 cells were more than 2 × 10^−6^ cm/s (high permeability), 0.2–2 × 10^−6^ cm/s (intermediate permeability), and less than 0.2 × 10^−6^ cm/s (low permeability). Using these criteria, it is clear from Table 1 that the majority of the compounds studied in 2/4/A1 and Caco-2 monolayers were highly permeable.

**Figure 1:**
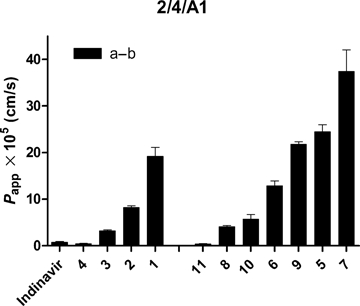
Transport of HIV-1 protease inhibitors in 2/4/A1 cell monolayers. The transport was studied in apical-to-basolateral (a–b) direction as described in *Materials and Methods*. BSA (1%) was present in the receiver solutions. Each bar represents the mean of permeability coefficient (*P*_app_) ± SD, *n* = 3.

A closer examination of the permeability data showed that the passive permeability of the PIs in 2/4/A1 cells covered a range from low to high. Thus, the *P*_app_ values ranged from 3.8 × 10^−6^ cm/s (compound **11**) to 374 × 10^−6^ cm/s (compound **7**), which is about a 100-fold variation (Table 1; Fig. 1). Surprisingly, no relationship could be observed between the permeability and activity in the cell assay (EC_50_).

In compounds **1–4**, the R_1_ substituent was the indanol used in this position in indinavir, whereas the R_2_ substituent was varied in size in the order bromo- <, phenyl- < styrene- < benzothiophene-. Interestingly, the permeability decreased with increasing size of the R_2_ substituent, and the permeability of the benzothiophene-substituted compound **4** was 47 times lower than that of the bromo-substituted compound (**1**) as in turn had 24 times higher than that of indinavir (Fig. 1).

In compounds **5–10**, the bromo substituent was kept constant in the R_2_ position, promoting high permeability as judged from compounds **1–4**. Instead, the R_1_ substituent was varied with the help of structural motifs taken, at least in part, from registered PIs. These included the tetrahydrofuryl substituent in compound **6** (amprenavir), the hydroxymethylphenyl in compound **7** (nelfinavir), and the *tert*-leucine–*N*-methyl amide in compounds **10** and **11** (atazanavir). In compound **11**, in addition, the R_1_ substituent was altered to 2-pyridyl (atazanavir). In compounds **8** and **9**, valine–*N*-methoxyethyl amide and phenylalanine–*N*-methyl amide were used as R_1_ substituents, forming a small series with side chains of neutral amino acids together with compound **10**. In compound **5**, a benzyl group was used as the substituent in position R_1_.

Although the passive permeabilities of compounds **5–10** were high, compound **11**, which mimics atazanavir also in the R_2_ position (2-pyridyl-substituent), had a low passive permeability, giving further support to the idea that the smaller bromo substituent in the R_2_ position promotes high permeability. Thus, for compounds **5–10**, the permeabilities ranged from 41 × 10^−6^ cm/s (compound **8**) to 374 × 10^−6^ cm/s (compound **7**), corresponding to a ninefold variation. Interestingly, compound **7** shares the hydroxymethylphenyl substituent with nelfinavir, a PI with good absorption, but with a highly variable bioavailability.^31^ The permeability of compound **9** carrying the phenylalanine–*N*-methyl amide substituent was approximately four times higher than those of compounds **8** (valine–*N*-methoxyethyl amide) and **10** (*tert*-leucine–*N*-methyl amide). Clearly, the larger aromatic phenylalanine side chain affords a higher permeability.

**Figure 2:**
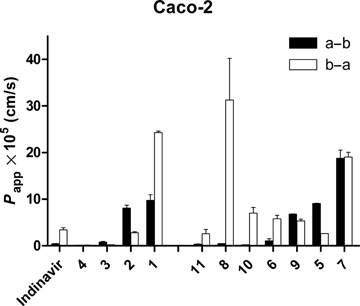
Transport of HIV-1 protease inhibitors in Caco-2 cell monolayers. The transport was studied in apical-to-basolateral (a–b) and basolateral-to-apical (b–a) directions as described in *Materials and Methods*. BSA (1%) was present in the receiver solutions. Each bar represents the mean of permeability coefficient (*P*_app_) ± SD, *n* = 3.

Because the discrepancy in the potency of the PIs between the cell-free and the cell assays could not be related to differences in passive permeability, we investigated the bidirectional permeability in Caco-2 cells (Table 1, Fig. 2). This approach allows the identification of active transport mechanisms, in particular, by ABC transporters such as P-gp.^13^,^32^ From the results in Caco-2 cells, a partly different picture emerges. Although the permeability ranking was qualitatively comparable to that obtained in 2/4/A1 cells, several of the compounds had large efflux components, resulting in low permeabilities in the absorptive (a–b) direction. For compounds **2** and **5**, a possible active uptake process was observed.

As in 2/4/A1 cells, indinavir displayed a relatively low permeability in the absorptive direction, whereas the permeability in the reverse secretory direction was eight times higher, which is related to the fact that indinavir is a substrate of P-gp, and is in agreement with the published data.^32^ In the indanol series, a significant efflux was also observed for compound **1**, which is the most likely reason for the permeability in the absorptive direction for this compound being half that observed in the 2/4/A1 cells, lacking functional P-gp. In contrast, compound **4**, with the benzothiophene substituent in the R_2_ position, did not exhibit a higher permeability in the secretory direction. It did, however, have the lowest permeability of compounds **1–4**, supporting the observation in the 2/4/A1 cells that R_2_ substitution with the bulky benzothiophene reduces the permeability.

For compounds **5–11**, the highest efflux ratios were observed for the *tert*-leucine–*N*-methyl amide R_1_-substituted compounds, compounds **10** and **11**, and in particular, the valine–*N*-methoxyethyl amide-substituted compound, compound **8**, the latter obtaining and efflux ratio of 65. This is in sharp contrast to the highly permeable compound **9**, carrying the phenylalanine–*N*-methyl amide, for which no efflux could be observed. This finding is in agreement with the literature, which indicates that rather small structural changes switch a P-gp substrate to form a nonsubstrate.^33^,^34^ As in the 2/4/A1 studies, in addition to compound **9**, the highest permeabilities in the absorptive direction were observed for compounds **5** and **7**, that is, the two compounds with the smallest aromatic substituents in the R_1_ position. Of particular interest is the fact that compound **7** did not show any efflux at all. As for the passive permeability data, the observed differences in ABC transporter-mediated efflux in Caco-2 cells could not explain the discrepancy in potency in the cell-free and cellular assays for the PI series.

## DISCUSSION

At the onset of this study, we wanted to investigate whether 2/4/A1 cell monolayers offer a suitable model for studies of passive drug transport in drug discovery and, more specifically, for ranking of structural leads according to their permeability. For this purpose, we used a series of HIV-1 PIs because these compounds present complex pharmacokinetic issues, and limited information is available regarding their structure–permeability and structure–efflux properties. In addition, compounds from the selected PI series displayed a large discrepancy in the inhibitory potential between the cell-free activity assay, wherein many compounds were active in the nanomolar range, and the activity in a cellular assay, wherein the PI needs to permeate the cell membrane prior to reaching its target and wherein the compounds could be inactive in concentrations up to 10 µM. Because the compounds were considered to have good to acceptable metabolic stability,^18^ we speculated that permeability and efflux studies could explain this issue.

Our results clearly indicate that permeability experiments in 2/4/A1 cell monolayers differentiate between passively transported compounds with pharmacokinetic liabilities, as exemplified by the HIV-1 PIs. Advantages of 2/4/A1 cells include a tight junction pore distribution that corresponds better with those in the human small intestine than Caco-2 cells,^5^ resulting in quantitatively comparable permeability data to those obtained in the perfused human intestine in conscious humans^4^,^7^ and the absence of measurably active drug transport processes, which enables studies of passive permeability in isolation to be made.^8^ However, comparative efflux studies in Caco-2 cells revealed that some of the highly permeable compounds in 2/4/A1 monolayers had significant efflux components, reducing the permeability in the absorptive direction from the high to the intermediate range. Importantly, because the PIs in this investigation were uncharged at the physiological pH, they were not subject to charge-dependent pH traps such as lysosomal accumulation that may mimic active transport.^35^,^36^

In drug discovery, an efflux ratio larger than two is often used as a warning of the likelihood of a significant drug efflux^37^ and thereby indicating compounds warranting additional *in vitro* studies.^38^ Much larger ratios were observed for some of the compounds in our study, pointing to a need for the routine investigation of ABC transporter-mediated efflux of PIs in early drug discovery. Several conclusions can be drawn from this study regarding how the substituent structure and position can have an impact on the passive permeability and active efflux, respectively. First, a small substituent, such as a bromo group in the R_2_ position, seems to promote high permeability. The larger the substituent in this position, the lower the permeability. Second, the hydroxymethylphenyl substituent in the R_1_ position both enhanced the permeability and limited the efflux, at least for the bromo-substituted compound **7**. Another small aromatic benzyl residue in this position had a similar effect. Third, R_1_ substituents containing amide-substituted hydrophobic amino acids were good substrates for efflux transporters in Caco-2 cells, most likely through P-gp. This resulted in a 23- and ninefold (for leucine- and valine-containing compounds **10** and **8**, respectively) reduction in permeability in the absorptive direction in Caco-2 cells in comparison with 2/4/A1 cells. However, when the amino acid was changed to the aromatic phenylalanine, the permeability was increased and the efflux was abolished. The higher uptake of compounds **3** and **5** observed in the absorptive direction requires further investigation before an active mechanism can be confirmed and eventually identified. Caco-2 cells express several functional uptake transporters in their apical membranes, but an investigation of their role in the uptake of compounds **3** and **5** was beyond the scope of this paper.

Thus, for good and for bad, Caco-2 cell monolayers provide a permeability coefficient that is a mixture of passive and active transport processes. Although this may serve as a warning bell for drug efflux, as in this study, it should be pointed out that the variable expression of transport proteins in Caco-2 monolayers may result in over- or underinterpretation of such data.^39^

Partial least-square analysis of the passive permeability data in 2/4/A1 cells was also performed to obtain further information about the structural requirements for high and low permeability (see supplementary information, S2). Two models were built: Model 1 is based on descriptors calculated for the entire molecule and model 2 is based on descriptors for the substituent positions R_1_ and R_2_, respectively. Model 2 was somewhat more predictive, as judged by the reported *Q*^2^ values. Model 2 was composed of two latent variables obtained from nine descriptors with a significant statistical quality and a cross-validated *Q*^2^ value of 0.88. The model supported the analysis performed above. Properties related to the aromatic character of the R_1_ substituent, aromatic ratio, and number of aromatic bonds were important for enhancing the transport across the 2/4/A1 cells. The R_2_ substituent should be as small as possible to promote transport across the cells, as indicated by variables such as the “number of non-H atoms” and the “number of bonds”. This was further manifested by the negative coefficients for all the descriptors of this substituent (R_2_). Modeling of efflux was not performed because this response was not “pure,” as the contribution of various transport pathways could vary from one compound to another. Computational modeling of P-gp interactions is possible using other systems, wherein the transport protein is overexpressed and the expression of endogenous transporters is kept at background levels.^40^

Our investigation did not provide an explanation for the discrepancy in the PI activity between the cell-free assays and the cellular ones. Initially, we investigated drug uptake in the MT4 cell line used in the cellular anti-HIV activity assay. Unfortunately, the results were not conclusive with regard to positive controls for P-gp transport (data not shown), and we therefore turned to cell systems that perform reliably in our laboratory. However, our hypothesis that variation in membrane permeability or active drug efflux in intestinal epithelial monolayers could provide clues about the different results in the efficacy assays turned out to be false. Recent studies indicate that there are significant differences in the transport protein expression in various cell lines used in drug discovery.^41^ Furthermore, the expression of efflux proteins in HIV-1-infected MT4 cells used in the cellular assay of PI activity could be quite different from that in uninfected MT4 cells and in the Caco-2 cells used in the present study. In addition, the nonspecific binding of the PIs in HIV-infected cells may be different from that in healthy cells. It would therefore be interesting to study the transporter expression and function of HIV-infected MT4 cells, but such studies were out of the scope of the present study. Nevertheless, our investigation provided important insights into structural features influencing the active efflux and passive transport of the PIs and, hopefully, these results can be used to improve the membrane permeability and to reduce the efflux of new series of HIV PIs.

## CONCLUSIONS

2/4/A1 cells ranked passive permeability in a broad range that allowed the identification of structural elements that either enhanced or reduced the passive permeability of the investigated series of HIV PIs. Thus, in particular, a small aromatic R_1_ substituent and a small (bromo-) R_2_ substituent were related to a high permeability. Similarly, bidirectional studies in Caco-2 cells associated amide-substituted small neutral hydrophobic amino acids, such as valine and leucine, in the R_1_ position with enhanced efflux, whereas the corresponding phenyl-*N*-methyl amide was found to enhance permeability and to abolish efflux. We hope that these findings will be useful for the search for HIV-1 PIs with improved pharmacokinetic and dynamic properties.

## Abbreviations used

PIs: protease inhibitors
BSA: bovine serum albumin
ECM: extracellular matrix
DMSO: dimethyl sulfoxide
HBSS: Hanks' balanced salts solution
EGF: epidermal growth factor
HEPES: 4-(2-hydroxyethyl)-1-piperazineethanesulfonic acid
*P*_app_: apparent permeability coefficient
TEER: transepithelial electrical resistance
ABC transporters: ATP-binding cassette transporters
LC–MS/MS: liquid chromatography–tandem mass spectrometry
TPSA: topological polar surface area
Clog *P*: calculated log octanol–water partition coefficient of unionized species, log *D*, calculated log distribution coefficient of unionized and ionized species in octanol–buffer (pH 7.4)
PLS: the partial least-squares projection to latent structures
*K*_i_: binding affinity (dissociation) constant
EC_50_: half maximal effective concentration
