# Supplementary material for: Structural Features Determining the Intestinal Epithelial Permeability and Efflux of Novel HIV-1 Protease Inhibitors
Source: J Pharm Sci. 2011 Apr 13;100(9):3763–72. doi: 10.1002/jps.22570 (PMC3210832; doi:10.1002/jps.22570)
Supplement: Supplementary file 1 [file jps0100-3763-SD1.doc]

# Supplementary Information

# Structural features determining the intestinal epithelial permeability and efflux of novel HIV-1 protease inhibitors

Lucia Lazorova ^a^, Ina Hubatsch ^a^, Jenny K. Ekegren ^b^, Johan Gising ^b^, Daisuke Nakai ^a d^, Noha Zaki ^a e^, Christel A. M. Bergström ^a^, Ulf Norinder ^a c^, Mats Larhed ^b^, Per Artursson ^a^ *

**Synthesis of methyl (S)-1-(2-((S)-2-benzyl-3-((S)-3,3-dimethyl-1-(methylamino)-1-oxobutan-2-ylamino)-2-hydroxy-3-oxopropyl)-2-(4-(pyridin-2-yl)benzyl)hydrazinyl)-3,3-dimethyl-1-oxobutan-2-ylcarbamate (11).**

A 2 mL smith vial was loaded with **10** [^1^](#_ENREF_1) (55 mg, 0.08 mmol), cupper(II) oxide (8 mg, 0.10 mmol), bis(triphenylphosphine)palladiumchloride (4 mg, 0.006 mmol), 2-(tributylstannyl)pyridine (123 mg, 0.33 mmol) and DMF (1.5 mL). The vial was capped and heated using microwaves at 130 °C for 20 min. DCM (5 mL) was added and the mixture was washed with saturated NaHCO_3_ (aq.) (3x10 mL). The organic layer was dried with MgSO_4_ and evaporated. The residue was in MeCN (4 mL) and washed with iso-hexane (3x10 mL). The MeCN layer was concentrated and the product purified by reverse-phase HPLC (30 min gradient of 15-60% MeCN in 0.05% aqueous formic acid) to afford **11** (7 mg, 13% yield) as a white solid. [α]_D_^22^ = -63,2° (*c* 0.53, CD_3_OD); ^1^H NMR (CD_3_OD) δ 8.58 (dm, *J* = 4.9 Hz, 1H), 7.87 (dm, *J* = 7.4 Hz, 1H), 7.83 (m, 2H), 7.80 (m, 1H), 7.49 (m, 2H), 7.34 (ddm, *J* = 7.4, 4.9 Hz, 1H), 7.20-7.14 (m, 5H), 4.17 (d, *J* = 14.5 Hz, 1H), 4.11 (d, *J* = 14.5 Hz, 1H), 3.89 (s, 1H), 3.78 (d, *J* = 14.0 Hz, 1H), 3.66 (s, 1H), 3.57 (s, 3H), 2.97 (d, *J* = 14.0 Hz, 1H), 2.89 (d, *J* = 13.3 Hz, 1H), 2.78 (d, *J* = 13.3 Hz, 1H), 2.58 (s, 3H), 0.80 (s, 9H), 0.64 (s, 9H); ^13^C NMR (CD_3_OD) δ 176.5, 172.4, 172.0, 159.0, 158.6, 150.3, 139.9, 139.6, 138.9, 137.1, 131.3, 130.1, 128.9, 128.0, 127.4, 123.7, 122.4, 79.1, 68.3, 63.0, 62.6, 62.0, 52.7, 44.6, 35.8, 35.0, 27.1, 26.7, 26.0; ESI-MS (*m/z*): 675 (M + H^+^); HRMS (M+H^+^): 675.3875, C_37_H_51_N_6_O_6_ requires 675.3870.

**HIV-1 protease inhibition of 11.**

*K*_i_ values for the synthesized compounds were determined from two individual measurements by a fluorometric assay using the fluorescent substrate DABCYL-γ-Abu-Ser-Gln-ASN-Tyr-Pro-Ile-Val-Gln-EDANS (Bachem, Bubendorf, Switzerland) as was described previously . More specifically, the *K*_i_ values for the synthesized compounds were determined from two individual measurements by a fluorometric assay using the fluorescent substrate DABCYL-γ-Abu-Ser-Gln-ASN-Tyr-Pro-Ile-Val-Gln-EDANS (Bachem, Bubendorf, Switzerland)[^3^](#_ENREF_3). Measurements were performed in 96-well plates with a Fluoroskan plate reader (Labsystems, Helsinki, Finland). The excitation and emission wavelengths were 355 and 500 nm, respectively. All incubations were performed at 30 ºC in 0.1 M sodium acetate-1 M NaCl-1 mM dithiothreitol (DTT)-1mM EDTA-3% DMSO at pH 5.0 with 5 µM substrate. With the intention of ensuring that the substrate and inhibitor would be dissolved completely, all components (300 µL) were preincubated for at least 20 min before the reaction was started by adding enzyme. The initial rates were measured over periods of 5 min. The data were analyzed by nonlinear regression using SIMFIT and an equation for tightly binding inhibitors. The kinetic constants (*k*_cat_ and *K_m_*) were determined from the spectrophotometric assay with a chromophoric peptide substrate. The substrate concentration was varied over as wide a range as the sensitivity and solubility limitations permitted. The kinetic constants were estimated by nonlinear regression analysis using SIMFIT and the equation for simple Michaelis-Menten kinetics. The assay variability was checked by the inclusion of a known inhibitor and the standard deviation for the enzyme assays was ±50% of the mean.

**In vitro anti-HIV-1 protease activity of 11.**

The in vitro anti-HIV activity (EC50 values) was assayed in MT4 cells using the colorimetric XTT assay to monitor the cytopathogenic effects according to a previously published procedure [^1-3^](#_ENREF_1) .

In more detail, stock solutions of phosphoramidate compounds were made by dissolving the inhibitors in dimethylsulfoxide (DMSO) to obtain a final concentration of 10 mg ml^-1^. MT4 cells were grown in RPMI 1640 cell culture medium, including 10% fetal calf serum and 2 mM glutamate, infected with 10 TCID_50_ HIV-1 per 2×10^4^ cells, and cultured in the presence of different concentrations of inhibitors for 6 days. The colorless reagent 2,3-bis[2-methoxy-4-nitro-5-sulphophenyl]-5-[(phenylamino)carbonyl]-2H-tetrazolium hydroxide (XTT) was added and the amount of XTT-formazan produced by metabolic conversion in viable cells during the next 6 h was measured at 450 nm. Mutant HIV was obtained by passaging cell-free virus in the presence of stepwise increased concentrations of a saquinavir, symmetric diol based inhibitor[^4^](#_ENREF_4) and ritonavir as described in the literature[^3^](#_ENREF_3).

# S 1:

| Model | R^2^ | Q^2^ | RSD | N | Comp |
| --- | --- | --- | --- | --- | --- |
| 1 | 0.850 | 0.630 | 0.799 | 12 | 3 |
| 2 | 0.910 | 0.880 | 0.605 | 11 | 2 |

R^2^: squared correlation coefficient, Q^2^: cross-validated correlation coefficient, RSD: residual standard deviation, N: number of compounds, Comp: number of PLS components

## S 2: Observed and predicted values for models 1 – 2

| **Obs ID (Primary)** | **SMILES** | **Experimental sqrt_papp_24a1** | **Model 1 Predicted sqrt_papp_24a1** | **Model 2 Predicted sqrt_papp_24a1** |
| --- | --- | --- | --- | --- |
| **11** | CNC([C@H](C(C)(C)C)NC([C@@](CC1=CC=CC=C1)(O)CN(CC2=CC=C(C3=NC=CC=C3)C=C2)NC([C@H](C(C)C)NC(OC)=O)=O)=O)=O | 0.620 | 0.759 | 0.410 |
| **Indinavir** | CC(C)(C)NC(=O)C1CN(CCN1CC(O)CC(Cc2ccccc2)C(=O)NC3C(O)Cc4ccccc34)Cc5cccnc5 | 0.887 | 0.684 |  |
| **1** | c1(ccc(cc1)Br)CN(C[C@](C(N[C@@H]3[C@@H](Cc2ccccc23)O)=O)(Cc4ccccc4)O)NC([C@H](NC(OC)=O)C(C)(C)C)=O | 4.385 | 4.689 | 4.887 |
| **3** | c1(ccc(cc1)\C=C\c2ccccc2)CN(C[C@](C(N[C@@H]4[C@@H](Cc3ccccc34)O)=O)(Cc5ccccc5)O)NC([C@H](NC(OC)=O)C(C)(C)C)=O | 1.789 | 2.544 | 1.799 |
| **4** | c1(ccc(cc1)c2cc3c(s2)cccc3)CN(C[C@](C(N[C@@H]5[C@@H](Cc4ccccc45)O)=O)(Cc6ccccc6)O)NC([C@H](NC(OC)=O)C(C)(C)C)=O | 0.643 | 0.849 | 0.977 |
| **2** | c1(ccc(cc1)c2ccccc2)CN(C[C@](C(N[C@@H]4[C@@H](Cc3ccccc34)O)=O)(Cc5ccccc5)O)NC([C@H](NC(OC)=O)C(C)(C)C)=O | 2.868 | 2.472 | 2.508 |
| **5** | C(NC([C@@](CN(NC([C@@H](NC(OC)=O)C(C)(C)C)=O)Cc1ccc(cc1)Br)(O)Cc2ccccc2)=O)c3ccccc3 | 4.950 | 4.753 | 5.313 |
| **7** | N(C([C@@](CN(NC([C@@H](NC(OC)=O)C(C)(C)C)=O)Cc1ccc(cc1)Br)(O)Cc2ccccc2)=O)c3c(c(ccc3)O)C | 6.116 | 4.731 | 5.176 |
| **8** | N(C([C@@](CN(NC([C@@H](NC(OC)=O)C(C)(C)C)=O)Cc1ccc(cc1)Br)(O)Cc2ccccc2)=O)[C@@H](C(C)C)C(NCCOC)=O | 2.023 | 2.996 | 2.721 |
| **10** | N(C([C@@](CN(NC([C@@H](NC(OC)=O)C(C)(C)C)=O)Cc1ccc(cc1)Br)(O)Cc2ccccc2)=O)[C@@H](C(C)(C)C)C(NC)=O | 2.395 | 3.039 | 2.721 |
| **6** | N(C([C@@](CN(NC([C@@H](NC(OC)=O)C(C)(C)C)=O)Cc1ccc(cc1)Br)(O)Cc2ccccc2)=O)C3COCC3 | 3.594 | 4.144 | 2.721 |
| **9** | N(C([C@@](CN(NC([C@@H](NC(OC)=O)C(C)(C)C)=O)Cc1ccc(cc1)Br)(O)Cc2ccccc2)=O)[C@@H](Cc3ccccc3)C(NC)=O | 4.665 | 4.306 | 4.814 |

# References

1. Ekegren JK, Gising J, Wallberg H, Larhed M, Samuelsson B, Hallberg A 2006. Variations of the P2 group in HIV-1 protease inhibitors containing a tertiary alcohol in the transition-state mimicking scaffold. Org Biomol Chem 4(16):3040-3043.

2. Ekegren JK, Unge T, Safa MZ, Wallberg H, Samuelsson B, Hallberg A 2005. A new class of HIV-1 protease inhibitors containing a tertiary alcohol in the transition-state mimicking scaffold. J Med Chem 48(25):8098-8102.

3. Nillroth U, Vrang L, Markgren PO, Hulten J, Hallberg A, Danielson UH 1997. Human immunodeficiency virus type 1 proteinase resistance to symmetric cyclic urea inhibitor analogs. Antimicrob Agents Chemother 41(11):2383-2388.

4. Alterman M, Bjorsne M, Muhlman A, Classon B, Kvarnstrom I, Danielson H, Markgren PO, Nillroth U, Unge T, Hallberg A, Samuelsson B 1998. Design and synthesis of new potent C2-symmetric HIV-1 protease inhibitors. Use of L-mannaric acid as a peptidomimetic scaffold. J Med Chem 41(20):3782-3792.
